# Genetic and behavioural architecture of childhood eating behaviour and links to obesity risk

**DOI:** 10.64898/2026.09.02.26362007

**Published:** 2026-09-04

**Authors:** R Karimi, M Baur, GM Power, J Sundfjord, N Fragoso-Bargas, L Clement, OA Andreassen, G Davey Smith, PR Njølstad, RE Brandlistuen, H Ask, G Hemani, KK Ong, Z Kutalik, A Havdahl, M Vaudel, S Johansson

## Abstract

**Background/Objectives:**

Childhood appetitive traits are heritable behavioural phenotypes hypothesized to link genetic susceptibility to obesity risk. Yet their genetic architecture and role in mediating polygenic adiposity risk remain poorly understood.

**Methods:**

We conducted the largest survey of childhood eating behaviour to date, allowing us to perform genome-wide association studies of six appetitive domains derived from 18 items of the parent-reported Children’s Eating Behaviour Questionnaire in up to 31,018 eight-year-old children from the Norwegian Mother, Father and Child Cohort Study (MoBa). A trio-based design enabled decomposition of direct and indirect genetic effects on appetite and BMI.

**Results:**

We identified ten independent genome-wide significant loci for childhood eating behaviour, primarily across *Food Responsiveness* , *Satiety Responsiveness* , and *Food Fussiness* , eight of which lie at established childhood or adult BMI loci. *Food Responsiveness* and *Satiety Responsiveness* showed both phenotypic and genetic correlations with BMI trajectories from early childhood through adolescence. Statistical mediation analyses indicated that 22.1% and 10.4% of the aggregated genetic association with BMI at age 8 could be decomposed through these traits, respectively. Locus-specific patterns further suggested mechanistic pathways, with the *FTO* locus acting predominantly via *Food Responsiveness* , and the *ADCY3* locus via *Satiety Responsiveness* . Trio analyses demonstrated that both BMI and eating behaviour associations were predominantly explained by children’s inherited alleles, with minimal contribution from indirect effect from parental adiposity, although parental genetic liability influenced reporting of *Satiety Responsiveness* .

**Conclusions:**

Childhood appetitive traits capture a substantial proportion of genetic susceptibility to adiposity through distinct eating behaviour pathways (under standard mediation assumptions). These effects are primarily driven by the child’s own genotype rather than indirect parental influences, positioning appetite as a plausible, biologically grounded target for early obesity prevention.

## Full Text Availability

The license terms selected by the author(s) for this preprint version do not permit archiving in PMC. The full text is available from the preprint server.

